# *Pseudomonas aeruginosa* in the Early Childhood: A Case Report

**DOI:** 10.4137/ccrep.s713

**Published:** 2008-05-07

**Authors:** Flávia dos Santos Moraes, Andréa Gonçalves Antonio, Marta Lua Pimentel Winz Almeida, Rodolfo de Almeida Lima Castro, Roberto Vianna

**Affiliations:** 1 Postgraduate of Pediatric Dentistry at Veiga de Almeida University, Rio de Janeiro, Brazil.; 2 Professor of Pediatric Dentistry at Veiga de Almeida University, Rio de Janeiro, Brazil, Professor of Pediatric Dentistry at Veiga de Almeida University, Rio de Janeiro, Brazil.

**Keywords:** *Pseudomonas aeruginosa*, children, infection, dental development disorder

## Abstract

*Pseudomonas aeruginosa* is an opportunistic bacterium that usually affects immunocompromised patients, causing infections whose signals and symptoms are related to the affected organ. The patient presented in this article was infected when he was 9 months old. Such condition led to certain alterations like dental improperly positioned teeth, retained deciduous teeth, hipodonty of permanent teeth, atrophy of the upper jaw and dental crowding. Therefore, the purpose of this article is to report the case of a patient affected by *Pseudomonas aeruginosa* infection in the early childhood and to describe the dental development disorders as consequence of this fact.

## Introduction

The genus Pseudomonas comprehends gram-negative microorganisms with more than 100 species. The most frequent species is the *Pseudomonas aeruginosa*, which is responsible for 70% of infections of this genus [1,2]. It is an invasive and opportunistic microorganism, since it causes infections in human beings whose defenses are altered. Immunocompromised, recently operated and burned patients in hospitals are susceptible hosts of this kind of infection [3,4]. However, this bacillus can also be found in the skin, throat and intestinal flora of healthy individuals [1,5,6].

In the majority of the infections caused by *Pseudomonas aeruginosa*, the signals and symptoms are unspecific and related to the affected organ. This pathogen penetrates the affected skin or mucosa, invades them locally and produces systemic infection. It can also cause necrotizing pneumonia through contaminated respiratory equipment, necrotizing external otitis in elderly diabetic patients; ocular infection after surgical procedures or wounds; urinary tract infections through the insertion of catheters or irrigation solutions; meningitis after neurosurgery or endorcaditis after surgery or catheter usage [1]. Furthermore, infected wounds and burns produces a greenish-blue exudate which is fluorescent under ultraviolet light and may present a characteristic odor of grapefruit [1,2,7,8].

Once the bacterium has passed through the affected area, it can penetrate the bloodstream causing septicemia. In this case, it is classical the presence of skin hemorrhagic necrosis characterized by round erythematosus lesions, denominated ecthyma gangrenosum, which can be occurred in 15% of *Pseudomonas aeruginosa* septcemia. Clinically, it starts as an inflammatory plaque, papule, or macule, reddish in color, edematous, and painful, which in less than 12–24 h develops into a hemorrhagic blister, vesicle pustule, or nodule with a narrow inflammatory rim [2,8–10].

Nowadays, *Pseudomonas aeruginosa* is responsible for approximately 50% of the mortality rates of patients with septicemia in hospitals. The cases of bacteremia due to this pathogen occurs in infected post-operatory wounds of 5% of patients who were considered healthy before surgeries, 17% in diabetic patients and more than 40% in neutropenic and cancer patients [11,12].

Although *Pseudomonas aeruginosa’s* infection frequently affects the urinary tract, respiratory system, skin and mucosa, causing damage to the affected organ, there are no reports of patients with oral manifestations as a result of this infection. Therefore, the purpose of this article is to report the case of a patient infected by *Pseudomonas aeruginosa* in early childhood.

## Case Report

An 11 year-old Brazilian boy was sent to the Pediatric Dentistry Clinic of Veiga de Almeida University, in Rio de Janeiro, Brazil. His main complaint, according to his mother was the improperly teeth positioning which was already compromising the aesthetics of the patient. The mother reported that the child had the *Pseudomonas aeruginosa* (PA) infection when he was 9 months old, although he was healthy by this time.

His medical history revealed that when he was 9 months old, some white lesions had been observed in the oral cavity, which had been diagnosed as Candidiasis by a pediatrician. A second medical evaluation took place on the same day in a hospital, when a swab of the lesion was done and only the presence of *Candida albicans* was verified. One week after, the child was sent to the hospital due to lesions in the oral cavity. He stayed in the hospital, where he contracted pneumonia and lost weight. During this period, the white lesions spread all over the oral cavity forming plaques. The final diagnosis, stated by the pediatrician from the mentioned hospital, was PA infection. Besides, it also caused ulcerated lesions in the palate, tongue and gum. The patient went through an antibiotic therapy with gentamicin and ciprofloxacin during 14 days. The patient stayed during one month in the hospital, when the pediatrician noticed the total recovery of him.

**Figure 1:**
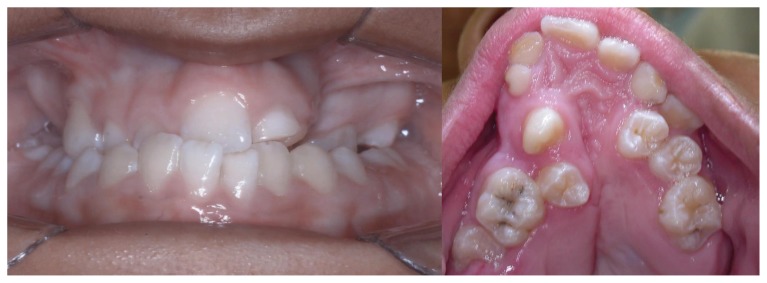
Frontal view, maxilla atrophy and longstanding retention of maxillary left central and lateral primary incisors.

**Figure 2:**
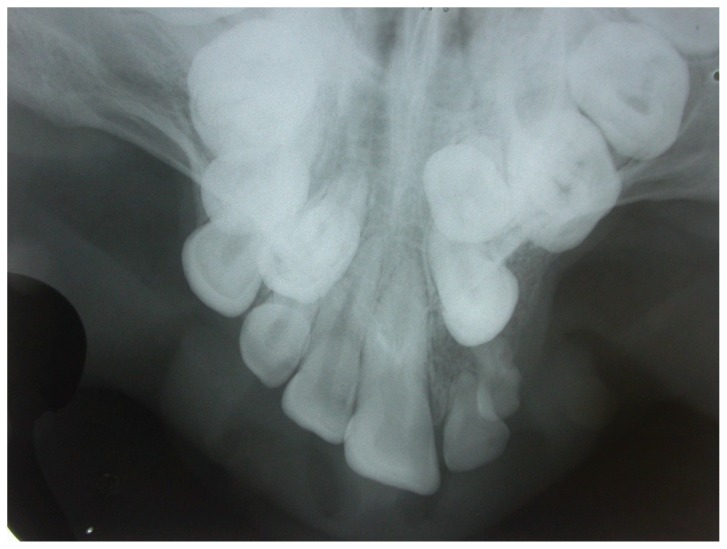
Hipodonty of maxillary left central and lateral primary incisors.

**Figure 3:**
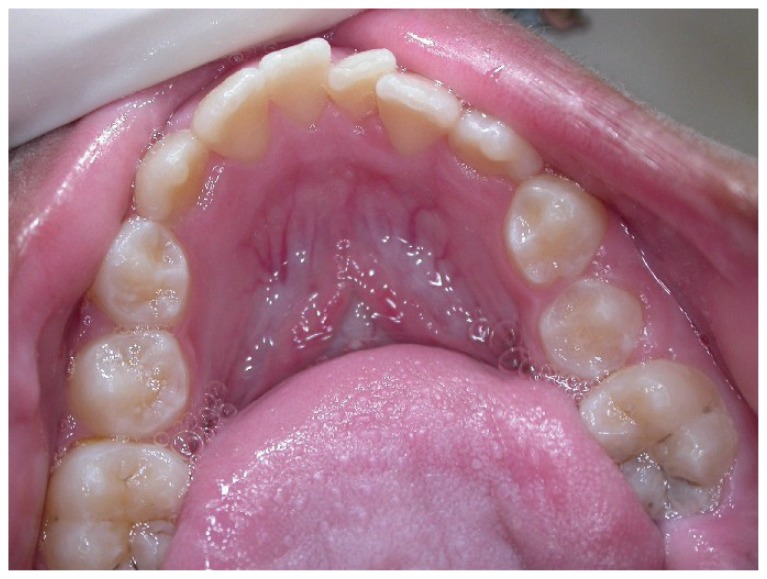
Crowding of mandibular inferior incisors.

Intra oral and radiographic examinations revealed: maxilla atrophy (Fig. 1), hipodonty of maxillary left central and lateral incisors and maxillary left first premolar (Fig. 2), longstanding retention of maxillary left central and lateral primary incisors, crowding of mandibular inferior incisors (Fig. 3), presence of maxillary right first and second premolar, left canine and left second premolar in the area of pre-maxilla. In addition, there were cavity lesions in the follow teeth: maxillary right first molar, maxillary right primary canine, maxillary left primary incisor, maxillary left lateral primary incisor, maxillary left first molar, mandibulary right and left first molars. Besides, it was noticed a disturbance in the patient speech.

After dental restorations are done, the patient will be sent to Orthodontics and Oral Maxillofacial Surgery for bone correction and malocclusion. The follow up will be done at every three months, considering the patient has a cariogenic diet and poor oral hygiene.

## Discussion

Despite being found in healthy individuals organs, PA causes infection and diseases, most commonly, in human beings whose defenses are altered by some other condition such as: patients in hospital with severe burns; pneumonia; immunodepressed patients because of chemotherapy or radiotherapy and chronic respiratory illnesses such as cystic fibrosis [2]. Nevertheless, the case reported differs from scientific literature, since the child has always good health and did not show any anomaly before being sent to hospital when he was 9 months old. Besides, another important fact to be considered is that the child went to hospital supposedly because of candidiasis and only after being hospitalized he was diagnosed with PA infection. Thus, the authors also mention the hypothesis that the child might have contracted such infection in the hospital.

Almost all body’s tissues are susceptible to PA infection. These bacteria are highly invasive and produce virulence factors that cause local tissue necrosis, fever, oliguria, hypotension, leukopenia or leukocytosis or disseminated intravascular coagulation and adult respiratory distress syndrome. In this report, the patient suffered ulcerated lesions in the palate, tongue and gum due to a PA infection, which probably resulted in necrosis, corroborating with what is found in literature [8,13] and consequently, the authors supposed that both bone and dental development were affected.

The diagnosis of PA infection is done through samples of cutaneous lesions, pus, urine, gob, among other materials that are submitted to laboratorial tests [2]. In the reported case, a swab of material from the oral cavity was done, however, the diagnosis of this infection had not been determined by this procedure, but only through laboratory exams such as culture of the ulcerate lesions, when the patient was interned in the hospital.

*Pseudomonas aeruginosa* can develop fast resistance to antibiotics, so it is not possible to combat with only one drug. It is recommended the realization of antibiograms in order to choose the best antibiotic therapy [1,2,7]. Actually, while the child was in hospital, gentamicin and ciprofloxacin were used in order to combat the infection. The antibiotics that are most mostly used in PA infection’s treatment are penicilins such as Piperacillin and Ticarcillin, aminoglycosides like Gentamicin and Amikacin, Imipenem, Quinolones, Aztreonam and Cephalosporines are also used [1,2,7,8,14,15]. Currently there is not a vaccine to protect against PA. Recently, intravenous imunoglobulin prophylactic used in high risk patients in the Intensive Care Unit, has reduced the case of PA septicemia [11]. Although the problem with PA infection has been resolved, the patient, who is 11 years old now, still has alterations in oral cavity, which jeopardizes his esthetics and speech. The authors believe that it could be directly related to what happened 10 years ago. These changes in the development probably happened because the patient was at an early stage of both bone and dental development and that wouldn’t have happened if the infection had taken place at a later time.

In this way, this report is extremely important to inform dentists and pediatric dentists of this condition and its possible consequences in the oral cavity.
